# Primordial Symptoms and ECG Among Sudden Cardiac Death Victims Due to Primary Myocardial Fibrosis

**DOI:** 10.1002/clc.70057

**Published:** 2025-07-01

**Authors:** H. Silvola, L. Pakanen, L. Holmström, J. S. Perkiömäki, M. A. E Eskuri, H. V. Huikuri, M. J. Junttila

**Affiliations:** ^1^ Research Unit of Internal Medicine Medical Research Center Oulu, Oulu University Hospital, University of Oulu Oulu Finland; ^2^ Forensic Medicine Unit Finnish Institute for Health and Welfare Oulu Finland; ^3^ Department of Forensic Medicine, Research Unit of Internal Medicine Medical Research Center Oulu, University of Oulu Oulu Finland

## Abstract

**Background:**

Sudden cardiac death (SCD) remains a major cause of death despite progress in prevention and intervention of cardiac diseases. The most common cause of nonischemic SCD in young individuals in Northern Finland is primary myocardial fibrosis (PMF).

**Methods:**

Fingesture study consists of 5869 prospectively collected subjects with SCD from Northern Finland collected from 1998 to 2017. Nonischemic etiology was the cause of SCD in 1477 (25%) subjects out of which primary myocardial fibrosis was the cause of SCD in 184 (12%) subjects (65% men, median age 55 ± 16 years). We examined the ante mortem ECG and medical history of the subjects to discover preceding symptoms and ECG changes.

**Results:**

Prior health care contact in electronic health record system (EHR) was found for 89 (48%) subjects and ECG was available for 52 (28%) subjects. Both medical history and ECG were available for 20 subjects (11%). We observed that transient loss of consciousness (TLOC) was the most common *symptom* recorded and was reported by 33 (37%) subjects. ECG was abnormal in 38 (73%) subjects. Fragmented QRS (fQRS) complex was found in 26 (50%) subjects. Vast majority, 87% of subjects had either TLOC or abnormal ECG. Only seven subjects with ECG or EHR history available had normal ECG and did not have TLOC.

**Conclusions:**

Many SCD victims with primary myocardial fibrosis had abnormal ECG or history of TLOC. The results suggest that the combination should generate careful cardiovascular examination to detect underlying myocardial disease and possibly prevent SCD.

## Graphical abstract

Patients with primary myocardial fibrosis (PMF) may be present with abnormal ECG and/or transient loss of consciousness. Especially the combination of the two should indicate the need for further medical examination.

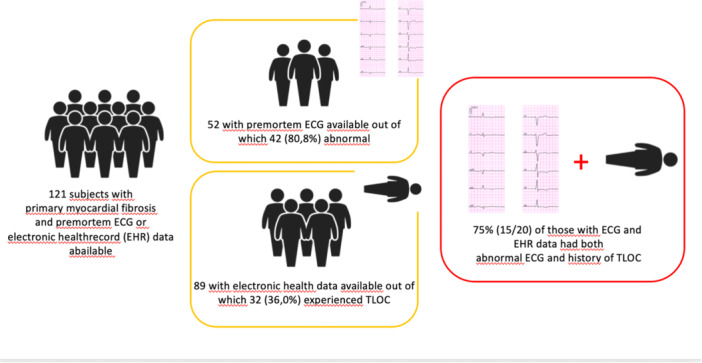

## 1 Introduction

Sudden cardiac death (SCD) remains a major cause of death despite decades of progress in prevention and intervention of cardiac diseases. *Up to 50% of SCDs are first cardiac events occurring in the absence of previously identified cardiac disease* [1]. Previous studies have shown that relative proportion of nonischaemic causes of SCD has increased and SCD due to coronary artery disease has decreased [2]. In this category, all cardiomyopathies leading to SCD have myocardial fibrosis as a common denominator. Furthermore, as has been previously reported, the most common cause of nonischemic SCD in young individuals (< 40 years old) in Northern Finland is primary myocardial fibrosis (PMF) [3]. PMF is defined by the presence of interstitial, diffuse of patchy fibrotic replacement of myocytes in the absence of any other detectable cardiac abnormality *at autopsy*.

Causes of PMF remain largely unknown. We have previously reported that 10% of SCD subjects with PMF have either pathogenic or likely pathogenic variant in a myocardial structure‐coding gene [4]. All pathogenic/likely pathogenic variants detected were either directly associated with the structural abnormality or were null variants in regions of genes which are commonly mutated in patients with inherited cardiomyopathies. In other words, PMF seems to share genotype patterns and may represent a variable phenotypic expression of a wide spectrum of these specific inherited structural diseases. In majority of subjects, genetic base of PMF was not discovered and therefore PMF may alternatively represent an entirely separate clinical entity from inherited cardiomyopathies. To prevent SCD in this population, individuals at risk need to be identified. 12‐lead ECG and thorough clinical history may help in identifying individuals with PMF and at risk for SCD.

## 2 Materials and Methods

Fingesture study consists of 5869 systematically autopsied subjects with SCD from Northern Finland collected from 1998 to 2017 [4]. Medico‐legal autopsies have been carried out by experienced pathologists using contemporary guidelines for diagnosis of death. The hearts of those with SCD were meticulously examined, including heart weight and wall thickness measurements, extent of coronary artery disease, histological examinations, and identification and characterization of myocardial fibrosis. A toxicology investigation was performed if there was suspicion of exposure or autopsy findings were insufficient to define the cause of death. There were no other noncardiac organ changes or prior diseases that could have caused myocardial fibrosis. Determination of the cause of death was also contributed to by medical records and questionnaires sent to the next of kin. Electronic Health record systems (EHR) were used to identify possible preceding symptoms of cardiac origin. The way ECGs were analyzed for changes has been previously reported [5]. If multiple ECGs were present, the most recent one was used. The study complies with the Declaration of Helsinki and was approved by the Ethics Committee of the University of Oulu.

## 3 Results

**FIGURE 1:**
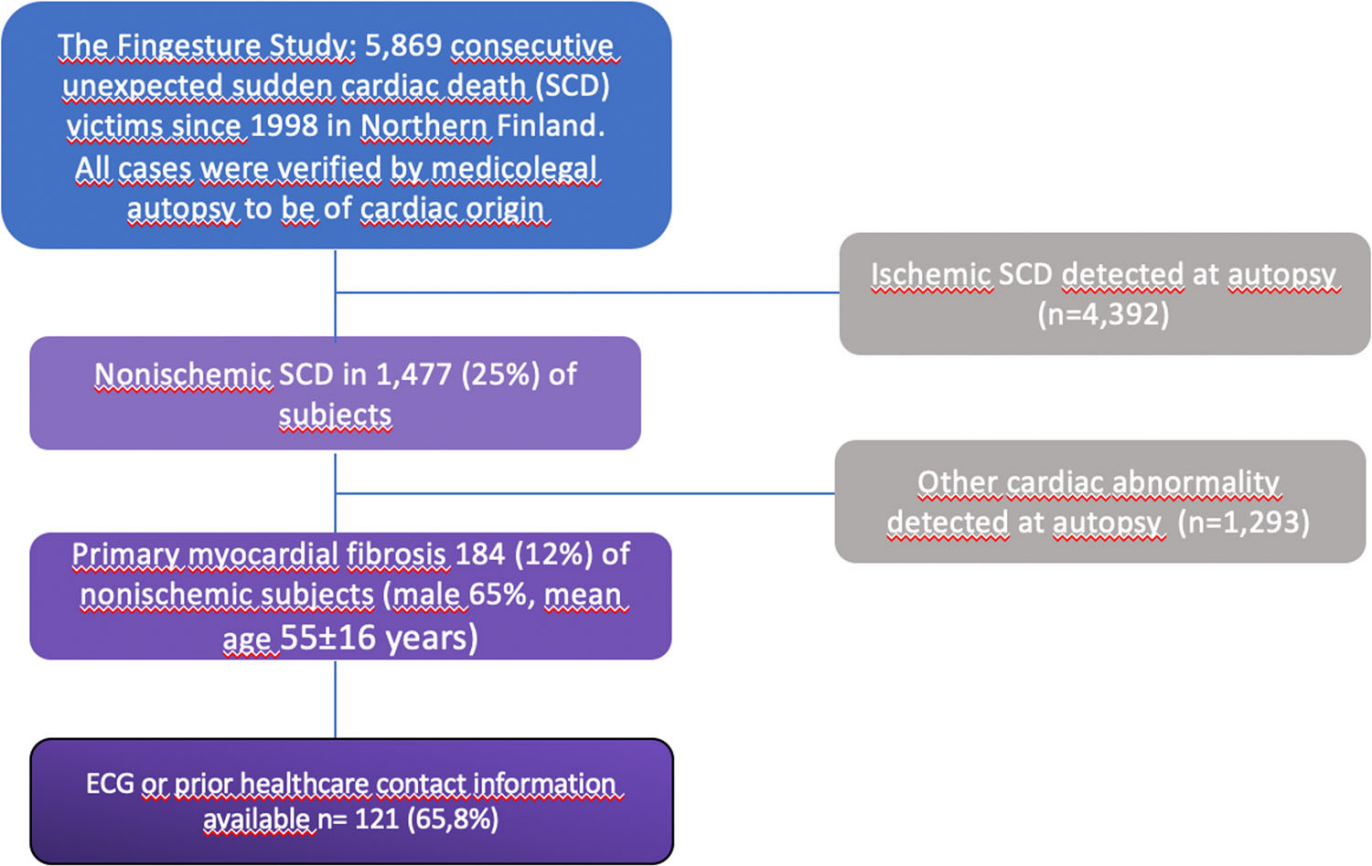
Selection of the study population.

In this study nonischemic etiology was the cause of SCD in 1477 (25%) subjects out of which primary myocardial fibrosis was the cause of SCD in 184 (12%) subjects (Figure 1). We examined the ante mortem ECG and medical history of the subjects to discover preceding symptoms and ECG changes. Electronic health record system (EHR) was searched for prior healthcare contacts and to obtain ECG. Most SCD victims with primary myocardial fibrosis were men 65% and median age was 55 ± 16 years even though PMF was relatively more common in women that in men. Prior health care contact in EHR was found for 89 (48%) subjects and prior ECG was available for 52 (28%) subjects; 20 subjects both medical history and ECG were available. We observed that transient loss of consciousness (TLOC) was the most common clinical *symptom* recorded and was reported by 33 (37% of those with EHR available, 18% of all) subjects. Other potentially relevant cardiac symptoms were remarkably rare.

**FIGURE 2:**
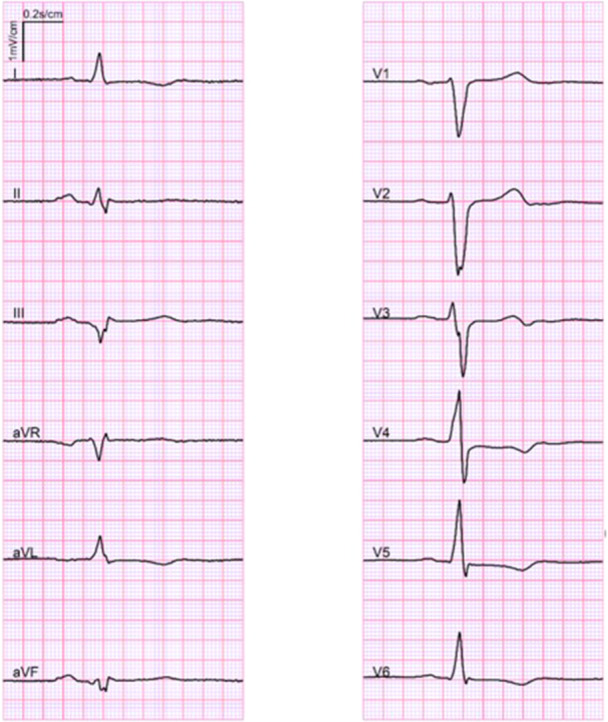
Electrocardiogram of a subject with sudden cardiac death due to primary myocardial fibrosis. This subject presented QRS fragmentation in leads III, aVL, aVF and V_3_. Also, T‐wave inversions were present in lateral leads. Paper speed 50 mm/s, gain 10 mm/mV.

ECG was abnormal in 38 (73% of those with ECG available and 21% of all) subjects (Figure 2). The most common abnormality was fragmented QRS complex (fQRS), which was found in 26 (50% of those with ECG available) subjects and was most common in inferior leads (23 subjects). Lateral or anterior fQRS was present in 16 (31%) subjects. Other ECG findings were prolonged QTc (> 450 ms in men and > 470 ms in women) in 10 (19%), T‐wave inversions (at least two in anterior, lateral and/or inferior leads) in 4 (8%), pathological Q‐waves in 3 (6%), and prolonged QRS (> 110 ms) in three subjects. Vast majority (87%) of those with ECG or EHR available, had either TLOC or abnormal ECG. Both were present in 15 individuals. Only seven subjects with ECG or EHR history available had normal ECG and did not have TLOC.

## 4 Discussion

This study demonstrates that warning signs may help to identify subjects with PMF at risk for SCD. Most feasible and accessible tool to raise suspicion of PMF may be the 12‐lead ECG and indeed it seems to be abnormal in most subjects. Fragmented QRS, T‐wave inversions, pathological Q‐waves, and prolonged QRS most probably represent myocardial fibrosis in this population [5]. In general population, prevalence of fQRS has been estimated to be 20% and 16% in inferior leads, which is far less than compared to our study population. Whether the type of fragmentation differs between those with myocardial fibrosis and those without remains undetermined so far. Prolonged QTc was also not an uncommon finding despite that it has not been associated with severity of myocardial fibrosis. In this study population prolonged QTc was secondary as none of the subjects had pathogenic or likely pathogenic variant in ion‐channel encoding gene. Whether prolonged QTc increases susceptibility to ventricular arrhythmias and sudden cardiac death in the context of PMF, is to be determined.

Apart from ECG, we found that transient loss of consciousness (TLOC) was identified as a potentially clinically relevant symptom, which was commonly reported by the subjects ante mortem. TLOC is a state of real or apparent loss of consciousness with loss of awareness, characterized by amnesia for the period of unconsciousness, abnormal motor control, loss of responsiveness, and short duration. Syncope is defined as TLOC due to cerebral hypoperfusion, characterized by rapid onset, short duration and spontaneous complete recovery. Retrospectively, it may be difficult to differentiate other forms of TLOC from syncope. Therefore, to count in all possible TLOC events of cardiac origin, we have included all non‐traumatic TLOC events in our analysis. None of the events was evaluated as neutrally mediated (reflex) syncope by the attending/treating physician.

It is commonly known that in approximately half of individuals, SCD is the first manifestation of the underlying heart disease [6]. Only about half of this PMF cohort had previous EHR available perhaps partly explained by the asymptomatic cardiac condition until SCD. PMF represents an entity without classical findings of cardiomyopathy but may be an alternative phenotype pathway or an early representation of cardiac structural disorders as we have previously reported [4]. Due to the nature of PMF, it may be challenging to identify individuals with this condition and at risk for SCD among the whole population.

## 5 Conclusions

This study shows that TLOC is common in SCD subjects with PMF possibly indicating prior history of arrhythmogenic syncope. Considering that many subjects had abnormal ECG or TLOC, at least the combination should generate careful cardiovascular examination including modern arrhythmia detection, evaluation of a possible substrate and finally, thorough risk stratification for SCD. From recognition of PMF as clinical entity and through systematic approach to assess risk, SCD may be preventable in this population.

## Conflicts of Interest

The authors declare no conflicts of interest.

## Data Availability

The data that support the findings of this study are available from the corresponding author upon reasonable request.

## References

[1] H. V. Huikuri , A. Castellanos , and R. J. Myerburg , “Sudden Death Due to Cardiac Arrhythmias,” New England Journal of Medicine 345, no. 20 (2001): 1473–1482. 11794197 10.1056/NEJMra000650

[2] M. J. Junttila , E. Hookana , K. S. Kaikkonen , M. L. Kortelainen , R. J. Myerburg , and H. V. Huikuri , “Temporal Trends in the Clinical and Pathological Characteristics of Victims of Sudden Cardiac Death in the Absence of Previously Identified Heart Disease,” Circulation: Arrhythmia and Electrophysiology 9, no. 6 (2016): e003723. 27301265 10.1161/CIRCEP.115.003723

[3] E. Hookana , M. J. Junttila , V. P. Puurunen , et al., “Causes of Nonischemic Sudden Cardiac Death in the Current Era,” Heart Rhythm: The Official Journal of the Heart Rhythm Society 8, no. 10 (2011): 1570–1575. 10.1016/j.hrthm.2011.06.031 21740887

[4] M. J. Junttila , L. Holmström , K. Pylkäs , et al., “Primary Myocardial Fibrosis as an Alternative Phenotype Pathway of Inherited Cardiac Structural Disorders,” Circulation 137, no. 25 (2018): 2716–2726. 29915098 10.1161/CIRCULATIONAHA.117.032175

[5] L. Holmström , A. Haukilahti , J. Vähätalo , et al., “Electrocardiographic Associations With Myocardial Fibrosis Among Sudden Cardiac Death Victims,” Heart 106, no. 13 (2020): 1001–1006. 32201371 10.1136/heartjnl-2019-316105

[6] E. Marijon , K. Narayanan , K. Smith , et al., “The Lancet Commission to Reduce the Global Burden of Sudden Cardiac Death: A Call for Multidisciplinary Action,” The Lancet 402, no. 10405 (2023): 883–936. 10.1016/S0140-6736(23)00875-9 37647926

